# Patient satisfaction and its influencing factors: results from a survey in inpatient department in a tertiary hospital setting in China

**DOI:** 10.1186/s12913-026-14238-2

**Published:** 2026-02-25

**Authors:** Cairu Xu, Xinpeng Xu, Haibo He, Yanfang Su, Jiansong Ji

**Affiliations:** 1 The Fifth Affiliated Hospital of Wenzhou Medical University, Lishui, Zhejiang 32300 China; 2 School of Public Health, Nanjing Medical University, Nanjing, 211166 China; 3 Evans School of Public Policy and Governance, University of Washington, Seattle, WA 98195 USA; 4 Wenzhou Medical University, Wenzhou, Zhejiang 325000 China

**Keywords:** Satisfaction, Ordered logistic regression, Inpatients, Influencing factors

## Abstract

**Background:**

Patient satisfaction constitutes one of the three dimensions of health system performance metrics, alongside health status and financial protection. However, a comprehensive understanding of the elements influencing inpatient satisfaction remains limited.

**Methods:**

This cross-sectional study evaluated inpatient satisfaction using a two-tiered analytical approach: an exploratory assessment of sociodemographic associations followed by a focused analysis of core service dimensions. A survey was conducted among 433 patients at the Fifth Affiliated Hospital of Wenzhou Medical University, employing a stratified random sampling technique. The Kruskal-Wallis test and ordered logistic regression were utilized to examine the relationships between inpatient satisfaction and its influencing factors, adjusting for sociodemographic variables.

**Results:**

The mean overall satisfaction score was 4.49. Satisfaction was highest in medical processes and technology, and lowest regarding costs. Univariate analysis identified payment method as a significant correlate. While exploratory univariate analysis of sociodemographic factors yielded limited significant associations, ordered logistic regression identified patients’ perceived quality across the five core service dimensions as the primary and significant predictors of overall inpatient satisfaction.

**Conclusions:**

Medical technology, doctor-patient communication, environmental factors, medical processes, and medical costs significantly and positively influence inpatient satisfaction. The overall satisfaction with inpatient services is affected by multiple factors, and enhancements can be achieved by optimizing doctor-patient communication, improving the hospital environment and facilities, and standardizing the medical service process.

**Supplementary Information:**

The online version contains supplementary material available at 10.1186/s12913-026-14238-2.

## Introduction

Inpatient satisfaction is a fundamental metric of healthcare service quality. Given the ongoing emphasis on service quality and patient experience within healthcare, patient satisfaction is a critical outcome measure of healthcare quality, directly influencing the frequency of medical disputes and hospital reputation. In recent years, the examination of inpatient satisfaction has emerged as a pivotal concern in healthcare quality improvement in the United States, the United Kingdom, Japan, and other countries [1].

International research on patient satisfaction has been conducted before, with the majority of studies employing standardized instruments and multivariate analysis [2–5]. The United States has prioritized healthcare quality research since the 1960s, establishing a national patient evaluation system through Agency for Healthcare Research and Quality in 1997, and introducing Hospital Consumer Assessment of Healthcare Providers and Systems in 2008 as a fundamental instrument for national healthcare management [6]; In 2002, the National Health Service Commissioning Board for Healthcare Quality in England conducted a patient satisfaction survey encompassing emergency, mental health, maternal and child health, and general practitioner services, subsequently relaying the findings to pertinent organizations to enhance healthcare services [7]. Consequently, relevant organizations in Japan, Korea, and other countries have initiated nationwide surveys regarding hospitalized patients’ satisfaction [8]. Chinese inpatient satisfaction research commenced in the late 1980s, initially characterized by a scarcity of measurement tools. Despite the proliferation of patient satisfaction surveys across various regions and hospitals in China, there remains an absence of standardized and unified questionnaires and evaluation systems. In the 21st century, the emergence of the patient experience concept has led to a gradual integration of satisfaction and experience assessments, with scale design increasingly emphasizing patient involvement and multifaceted service requirements [9–11]. For example, some study pointed out that with limited medical resources, hospitals could improve the quality of medical services by optimizing spatial distribution [12]. The Service Quality theory proposed by A. Parasuraman et al. delineates five dimensions of service quality that influence satisfaction: physical amenities, reliability, responsiveness, security, and emotional engagement [13]. Zhu and Ma asserted that satisfaction encompassed not only the comprehensiveness, safety, timeliness, and effectiveness of diagnosis and treatment, but also the quality dimensions inherent in the entire continuum of medical services rendered by healthcare professionals to patients [14]. The primary determinants of hospitalized patients’ satisfaction are the professional and technical competencies of medical personnel, doctor-patient communication, the hospital environment, and other facets of medical service delivery, while the impact of patients’ individual needs is comparatively minimal. Furthermore, while there is a lack of definitive literature illustrating that patient sociodemographic characteristics influence inpatient satisfaction, several studies have confirmed that such characteristics (e.g., age, gender, education level, income) do affect inpatient satisfaction, although the precise impact may differ based on the study population, region, and cultural context [15, 16].

In China, research on inpatient satisfaction has accumulated substantial empirical and theoretical insights. As early as 1989, Chen proposed that medical service activities fundamentally consist of two indispensable parties: the provider and the user, thereby framing satisfaction as an interactive outcome [17]. This foundational view has been subsequently refined and operationalized. For instance, Wu and Yu demonstrated that systematically assessing patient satisfaction based on the gap between expectations and actual experiences can reveal actionable weaknesses in service delivery [18]. Expanding the conceptualization of care quality, Deng distinguished between its technical aspects—which can be standardized and measured—and the non-technical dimensions, such as service attitude, communication, and environmental factors, which are directly perceived by patients [19]. This duality aligns with the multidimensional perspective later summarized by Li and Yan, who identified key influencing factors including the service attitude of healthcare professionals, doctor-patient communication, and medical technology [20]. Concurrently, health policy scholars like Xia et al. have advocated for a “people-oriented” approach and a quality control system centered on “patient focus and quality as the core,” highlighting the institutional imperative to align with patient-centered outcomes [21]. Consistently, recent work reinforces that patient satisfaction is a crucial indicator for evaluating service quality and a key driver for the high-quality development of public hospitals [22].Despite these contributions, several research gaps persist. While important dimensions have been identified, there is limited research that integrates sociodemographic variables, payment methods, and multi-dimensional service perceptions within a unified analytical model, particularly in the context of tertiary public hospitals serving mixed urban-rural populations. Many prior studies rely on descriptive or correlational analyses, with fewer employing advanced multivariate techniques, such as ordered logistic regression, to quantify the independent effects of different service dimensions while controlling for confounders. Findings regarding the influence of certain factors, remain inconsistent across studies, suggesting a need for context-specific clarification.

Given the persistent gap in standardized evaluation systems and the multifaceted nature of factors influencing inpatient satisfaction in China, this study aims to: describe the sociodemographic profile of inpatients at a tertiary public hospital in Zhejiang Province, China. Identify key service quality dimensions that significantly predict overall inpatient satisfaction, drawing on established theoretical frameworks. Quantitatively assess the impact of sociodemographic factors and service-related dimensions on inpatient satisfaction using a two-tiered analytical approach. Provide evidence-based recommendations for hospital management and policy-making to enhance service quality and patient experience in similar healthcare settings.

## Materials and methods

### Study setting

This study was conducted at the Fifth Affiliated Hospital of Wenzhou Medical University, located in Lishui City, Zhejiang Province, China. Lishui is a prefecture-level city in southwestern Zhejiang, characterized by a mixed urban and rural population. The hospital serves as a major tertiary medical center for the region, providing comprehensive healthcare services to residents of Lishui and neighboring areas. As a public tertiary hospital, it offers a wide range of medical specialties, including but not limited to internal medicine, surgery, orthopedics, obstetrics and gynecology, pediatrics, and emergency medicine. The hospital is equipped with advanced medical facilities and technologies, such as MRI, CT, digital subtraction angiography, and fully automated laboratory systems, supporting its clinical and diagnostic capabilities.The hospital has a total area of 202 acres, with over 4,000 staff members, including 1,052 party members, and 2,057 open beds. It serves over 2.3678 million outpatient and emergency visits annually, and admits approximately 104,300 inpatients each year. Additionally, the hospital performs about 41,000 surgical procedures annually, with an average length of stay of 6.35 days. It is organized into 142 departments and has consistently ranked within the top 5%–10% in the national tertiary public hospital performance evaluation for six consecutive years, achieving an A+ rating, with the highest ranking reaching 68th nationally. The patient population is diverse, encompassing urban residents, rural inhabitants, and individuals from various socioeconomic backgrounds, reflecting the demographic characteristics of the region.

### Survey design

The questionnaire was developed based on three theoretical frameworks: Maslow’s hierarchy of needs theory [23], the three-dimensional quality structure model [24], and Service Quality theory. These frameworks were systematically operationalized to guide the design of survey items and dimensions, ensuring a comprehensive assessment of factors influencing inpatient satisfaction.

Maslow’s hierarchy of needs informed items related to safety, comfort, and psychological support during hospitalization. Specifically, questions assessing ward cleanliness (item 19), nighttime quietness (item 20), empathetic communication (items 12–15), and food quality (item 18) were designed to capture patients’physiological, safety, and esteem-related needs. The three-dimensional quality structure model (structure–process–outcome) shaped the overall organization of the survey. structural elements included facility quality, medical equipment, and environmental amenities (items 9, 17, 21). Process elements covered doctor–patient communication, service procedures, waiting times, and admission/discharge efficiency (items 11–16, 22–25). Outcome perceptions were measured through overall satisfaction (item 30) and dimension-specific satisfaction scores. Service Quality theory was mapped onto the five core service dimensions evaluated in this study, aligning its five constructs—reliability, responsiveness, security, physical amenities, and empathy—with specific questionnaire items. Medical technology: reliability and security (items 7–10). Doctor–patient communication: responsiveness and empathy (items 11–16). Environment: physical amenities (items 17–21). Medical processes: reliability and responsiveness (items 22–25). Medical costs: security and transparency (items 26–29). A detailed mapping of theoretical constructs to individual survey items is provided in Supplementary Table S1. To enhance content validity and cultural relevance, the questionnaire also incorporated feedback from a panel of domestic and international healthcare experts and referenced China’s National Public Hospital Performance Evaluation Indicators. Furthermore, recognizing potential literacy or comprehension barriers among elderly respondents, investigators provided verbal clarification of items when necessary to support accurate and meaningful participation.

To further validate the questionnaire and survey procedure before the formal data collection, a pilot study was conducted. A convenience sample of 50 inpatients who met the inclusion criteria was recruited from the same hospital. Preliminary internal consistency was assessed using Cronbach’s α, which yielded a value of 0.955, indicating excellent reliability (α > 0.9). The pilot study confirmed the questionnaire’s acceptability and operational feasibility, supporting its use in the main survey.

### Data source

This study recruited inpatients from the Fifth Affiliated Hospital of Wenzhou Medical University from October to November 2024. We employed stratified random sampling to classify wards into 48 distinctive functional strata (e.g., internal medicine, surgery, orthopedics, obstetrics/gynecology). Ten hospitalized patients were randomly selected from each stratum, yielding 480 surveys participants. The inclusion criteria for the satisfaction survey were: (1) a hospitalization length of three days or more; and (2) provision of informed consent. The 3-day cutoff was chosen to ensure that patients had sufficient exposure to multiple aspects of inpatient care (e.g., nursing, environment, processes, and communication) to form a meaningful and stable assessment of satisfaction. Patients with shorter stays might not have experienced a full cycle of hospital services, which could introduce measurement noise. For inpatients who are incapable of independently completing questionnaires due to impaired consciousness, cognitive dysfunction, mobility limitations, or self-care disabilities, proxies, such as nurses or family caregivers facilitated the completion of the questionnaires. The questionnaire was prepared in accordance with China’s National Public Hospital Performance Evaluation Indicators [25]. An anonymous survey methodology was utilized to safeguard patient anonymity, minimize potential bias from external factors throughout the survey. The scale consists of two main elements: the socio-demographic characteristics of respondents and satisfaction metrics. The collected socio-demographic characteristics comprised of: (1) gender, (2) age, (3) educational attainment, (4) monthly income, (5) occupation, and (6) payment methods. The satisfaction survey evaluated five dimensions: (1) medical technology, (2) physician-patient communication [26], (3) environment, (4) clinical processes, and (5) healthcare costs. Data collection was conducted and administered electronically using the Questionnaire Star platform. Satisfaction levels across all dimensions and overall satisfaction are measured using a five-point Likert scale [27], with options including “very dissatisfied,” “somewhat dissatisfied,” “neutral,” “somewhat satisfied,” and “very satisfied.” Each option is assigned a score from 1 to 5, with 1 representing the lowest satisfaction level and 5 representing the highest satisfaction level. The scores for all items within a given dimension are summed, and the arithmetic mean is calculated. A higher score indicates higher satisfaction within that dimension.The self-administered questionnaire used in this study was developed by the authors to assess inpatient satisfaction across multiple domains (see Supplementary File S1).

### Statistical methods

Reliability analysis evaluates the internal consistency and stability of a measurement scale, determining whether all items consistently measure the same underlying construct.Validity analysis examines the extent to which a scale accurately measures the intended theoretical construct, including verification of its dimensional structure against hypothesized models. Correlation analysis quantifies the strength and direction of a monotonic association between two variables. Data cleaning procedures involved: (1) retaining only the most recent submission for duplicate questionnaires identified by the same mobile phone number or WeChat ID, (2) removing questionnaires exhibiting illogical response times, (3) removing questionnaires exhibiting illogical response patterns. Statistical analyses were performed utilizing SPSS 27.0. The level of satisfaction among respondents was characterized using descriptive statistics, including means with standard deviations for continuous variables and percentages for categorical variables. Reliability of the scale was assessed using Cronbach’s alpha coefficient (α), while structural validity was evaluated through the Kaiser-Meyer-Olkin (KMO) measure and Bartlett’s test of sphericity; A correlational analysis was conducted to investigate the relationships between the different dimensions of satisfaction and overall satisfaction [28]. Due to the non-normal distribution of satisfaction scores, three or more independent samples were analyzed using the Kruskal-Wallis H test for one-way analysis of socio-demographic characteristics, while two independent samples were analyzed with the Mann-Whitney U test for one-way analysis of significance, with a significance level of *p* < 0.05.Given that linear regression requires continuous variables and the dependent variable was an ordinal categorical variable, multivariate analyses were conducted using an ordered logistic regression model to identify determinants of patient satisfaction. To control for potential confounding effects, the model included sociodemographic characteristics (age, sex, education level, occupation, and payment method) as covariates, in addition to the five core service dimensions (medical technology, doctor-patient communication, environment, medical processes, and medical costs).

## Results

### Characteristics of the study sample

We collected data from 480 patients. After data cleansing, 433 valid survey responses were kept for data analysis, resulting in an effective recovery rate of 90.2%.

Among the surveyed inpatient patients, 239 (55.2%) were female. Female patients exhibited marginally lower satisfaction scores than their male counterparts. Individuals over 60 years accounted for the largest proportion (34.4%), followed by those aged 50–59 years at 20.8%. The percentage of patients under 20 years old was the lowest at 1.4%, although their satisfaction level was the greatest across all age groups. The proportion of patients with a middle school education or lower was the highest at 54.1%, whereas those with a master’s degree or more constituted the smallest group. Patients possessing a bachelor’s degree or higher exhibited the greatest levels of hospital satisfaction. The predominant income bracket was between 4,001 Yuan and 6,000 Yuan, whereas both high- and low-income groups were considerably smaller. Farmers compromised the largest segment of the occupational groups at 39.7%, whereas students constituted the smallest fraction at 2.8%. Concerning payment options, 80.8% of patients utilized urban and rural residents’ medical insurance, while the other patients relied on out-of-pocket payments or free medical service. Exploratory univariate analysis revealed that, aside from payment method, no statistically significant differences in overall satisfaction were detected across sociodemographic subgroups (Table 1). This suggests that these background characteristics alone are not strong independent predictors of satisfaction variance in this sample. The finding regarding payment method warrants further investigation.

**Table 1:** Summary statistics of hospitalized patients and results of Mann-Whitney U/Kruskal-Wallis *H tests*

Basic Information	Groups	*n* (%)	Satisfaction Score (Mean ± SD)	Z/H Score	*P*-value
Sex				-1.205	0.228
	Male	194 (44.8)	4.54 ± 0.846		
	Female	239 (55.2)	4.46 ± 0.887		
Age(years)				3.051	0.692
	≤ 20	6 (1.4)	4.83 ± 0.408		
	20–29	50 (11.5)	4.58 ± 0.758		
	30–39	60 (13.9)	4.32 ± 1.066		
	40–49	78 (18.0)	4.42 ± 0.947		
	50–59	90 (20.8)	4.59 ± 0.748		
	≥ 60	149 (34.4)	4.50 ± 0.851		
Education Level				4.848	0.183
	Middle school or below	237 (54.1)	4.51 ± 0.826		
	High school or secondary school	90 (20.8)	4.34 ± 0.996		
	Bachelor’s degree or college diploma	99 (22.9)	4.62 ± 0.765		
	Master’s degree or higher	7 (1.6)	4.00 ± 1.528		
monthly income(yuan)				3.822	0.481
	≤ 2000	86 (19.9)	4.59 ± 0.709		
	2001—4000	109 (25.2)	4.53 ± 0.845		
	4001—6000	119 (27.5)	4.51 ± 0.862		
	6001—8000	58 (13.4)	4.47 ± 0.941		
	≥ 8001	61 (14.1)	4.28 ± 1.035		
Occupation				9.199	0.101
	Students	12 (2.8)	4.67 ± 0.651		
	Corporate/ institutional employees	58 (13.4)	4.53 ± 0.863		
	Workers	45 (10.4)	4.60 ± 0.863		
	Self-employed individuals	77 (17.8)	4.29 ± 0.985		
	Farmers	172 (39.7)	4.47 ± 0.881		
	Others	69 (15.9)	4.65 ± 0.703		
Payment Methods				26.076	0.000
	Out-of-pocket	37 (8.5)	4.23 ± 1.146		
	Basic health insurance	350 (80.8)	4.50 ± 0.865		
	Free medical service	46 (10.6)	4.66 ± 0.635		

Note. Between-group comparisons were performed utilizing the Mann-Whitney *U* test for analyses involving two groups and the Kruskal-Wallis *H* test for three or more independent samples

### Reliability and exploratory factor analysis

The reliability and validity of the questionnaire was evaluated using Cronbach’s α coefficient and the Kaiser-Meyer-Olkin (KMO) measure. The Cronbach’s α values for the individual dimensions were 0.893, 0.872, 0.901, 0.874, and 0.850, respectively, with an overall α of 0.949, demonstrating strong reliability. The questionnaire data underwent exploratory factor analysis, yielding a KMO score of 0.935 and a significant Bartlett’s test of sphericity (χ² = 6838.470, *p* < 0.001), indicating the appropriateness for factor analysis.

### Correlation analysis of hospitalized patients’ satisfaction

Due to the variables’ nonconformity to normal distribution, Spearman’s rank correlation analysis was conducted. The results revealed statistically significant positive correlations between overall patient satisfaction and all dimensions of healthcare service quality (*p* < 0.01), with correlation coefficients ranging from 0.465 to 0.622, indicating moderate associations (Table 2). In social and health services research, such coefficients are considered meaningful and reflect that each dimension contributes independently to the overall evaluation.

**Table 2:** Spearman correlation analysis of patient satisfaction dimensions

Dimension	Medical Technology	Patient-Doctor Communication	Environmental	Medical Process	Medical Expenses
Overall satisfaction	0.516**	0.589**	0.622**	0.465**	0.597**

Note. *p* < 0.01 (two-tailed). Correlation coefficients were calculated using Spearman’s ρ

### Factors associated with patient satisfaction

To identify independent predictors of overall satisfaction while adjusting for sociodemographic factors, an ordered logistic regression model was constructed. The model included the five core service dimensions as primary predictors and controlled for age, sex, education level, occupation, and payment method based on their potential relevance. Multicollinearity diagnostics indicated tolerances exceeding 0.1 and variance inflation factors below 5, suggesting no severe multicollinearity. The parallel lines test yielded a p-value of 0.822 (> 0.05), confirming that the proportional odds assumption was met. The estimated results revealed that satisfaction with medical technology, doctor-patient communication, environment, medical processes, and medical costs are the main factors affecting inpatient satisfaction and have a significant effect on inpatient satisfaction. The regression results are shown in Table 3.

To assess the robustness of the associations, we compared a model containing only the five service dimensions (unadjusted model) with the final model that additionally adjusted for sociodemographic covariates (adjusted model). The direction and statistical significance of the odds ratios (ORs) for all five service dimensions remained consistent across both models. Specifically, the ORs for each dimension in the unadjusted model were as follows (Appendix Table S1): Medical Technology (OR = 1.766, *p* = 0.008), Doctor-Patient Communication (OR = 2.514, *p* < 0.001), Environment (OR = 2.941, *p* < 0.001), Medical Processes (OR = 1.690, *p* = 0.006), and Medical Expenses (OR = 1.948, *p* < 0.001). The inclusion of sociodemographic variables in the adjusted model (Table 3) resulted in only minor changes to these ORs (e.g., Medical Technology decreased from 1.766 to 1.618, while Environmental increased from 2.941 to 3.014), but did not alter their statistical significance or the substantive interpretation of the results. This consistency indicates that the positive associations between service quality perceptions and overall satisfaction are independent of the measured demographic background of patients.

**Table 3:** Ordered logistic regression analysis of factors associated with inpatient satisfaction, adjusted for sociodemographic variables

Factor	β	OR	*P*	Satisfaction Score (mean ± SD)	Tolerance	VIF
Medical Technology	0.481	1.618	0.026	4.37 ± 0.761	0.451	2.220
Patient-Doctor Communication	0.876	2.401	0.000	4.17 ± 0.815	0.389	2.568
Environmental	1.103	3.014	0.000	4.11 ± 0.841	0.465	2.151
Medical Process	0.621	1.861	0.001	4.35 ± 0.726	0.619	1.615
Medical Expenses	0.707	2.028	0.000	4.04 ± 0.911	0.522	1.915
Parallel-line test (*p*)	—	—	0.319	—	—	—

Note. The model included the five service dimensions as predictors and controlled for age, sex, education level, occupation, and payment method. β = standardized coefficient; OR = odds ratio; VIF = variance inflation factor. All p-values are two-tailed

The survey revealed that the average satisfaction score of inpatients at Wenzhou Medical University Fifth Affiliated Hospital was 4.49 ± 0.869, resulting in an overall satisfaction rate of 86.6%. The five dimensions of satisfaction including medical technology (OR = 1.618,*P* = 0.026), doctor-patient communication (OR = 2.401,*P* < 0.001), environmental factors (OR = 3.014,*P* < 0.001), medical processes (OR = 1.861,*P* = 0.001), and medical costs (OR = 2.028,*P* < 0.001) significantly influence inpatient satisfaction.

## Discussion

In comparison with prior research, the overall satisfaction score identified in this study was 4.49 ± 0.869, which aligns closely with benchmarks from large-scale national surveys. This indicates a consistently high baseline level of satisfaction in similar settings, and such consistency underscores the reliability of both the sample and the measurement approach.Moreover, unlike many earlier studies that relied primarily on descriptive statistics or correlation analyses, this study employed a two-tier analytical approach: exploratory univariate analysis followed by multivariate ordered logistic regression. This allowed us to disentangle the complex interplay among variables. While univariate analysis confirmed previous reports of a significant association between payment method and satisfaction, the multivariate model revealed that this association was attenuated when the five core service dimensions were included. This suggests that perceptions of care quality are more proximate determinants of overall satisfaction than sociodemographic characteristics alone—a nuance often overlooked in studies using simpler analytical methods.The study also found that the medical expenses dimension received the lowest satisfaction score among the five dimensions (4.04 ± 0.911). Regression analysis indicated that this score was a significant positive predictor of overall satisfaction (β = 0.692, OR = 1.997, *p* < 0.001), meaning that evaluation of costs directly influences overall evaluation. Consequently, its currently low rating constitutes a major bottleneck to improving overall satisfaction, highlighting the persistent systemic challenge of cost reasonableness.

### Considerations regarding the analytical approach

This study employed a two-tiered analytical strategy to understand the determinants of inpatient satisfaction. The initial exploratory analysis of sociodemographic characteristics served a descriptive purpose, providing a profile of the respondent sample. Therefore, the core of our analysis intentionally shifted to modeling the direct impact of five key service quality dimensions derived from established theory. The finding that most demographic variables showed no significant direct association with overall satisfaction, while all service dimensions emerged as strong predictors (Tables 1 and 3), reinforces the rationale for this focus. It underscores that patient satisfaction is more proximately and powerfully determined by perceptions of the received care—such as communication clarity, environmental comfort, and procedural efficiency—than by background characteristics. These service dimensions represent actionable levers for hospital quality improvement. Furthermore, the ordered logistic regression analysis adjusted for key sociodemographic variables (age, sex, education, occupation, and payment method), confirming that the associations between service dimensions and satisfaction remained robust even after accounting for these background characteristics. This underscores that the identified service factors are independent predictors of satisfaction, not merely reflections of patient demographics.To directly address the potential influence of background characteristics, we compared the regression model adjusting for sociodemographic covariates with an unadjusted model containing only the five service dimensions. This comparison confirmed that the strong, positive associations between all five service dimensions and overall satisfaction were not materially confounded by age, sex, education, occupation, or payment method. The stability of the effect estimates across both models reinforces the conclusion that perceptions of received care are the primary drivers of satisfaction in this setting.

The research indicated that there is a significant correlation between payment methods and overall satisfaction. Univariate analysis revealed that payment method was the only sociodemographic factor significantly associated with overall satisfaction, with patients utilizing “free medical service” reporting the highest satisfaction scores (4.66 ± 0.635) and those paying “out-of-pocket” exhibiting the lowest (4.23 ± 1.146). This finding warrants further interpretation, as it reflects the complex interplay between financial mechanisms, patient expectations, and perceived service quality within the Chinese healthcare context.Several interrelated factors may explain this disparity. First, perceived financial burden directly influences patient experience. Out-of-pocket patients bear the full cost of treatment, which may heighten their scrutiny of service quality and cost transparency. Any perceived inadequacies in these areas can amplify dissatisfaction. In contrast, patients under free medical schemes or comprehensive insurance experience reduced personal financial pressure, allowing them to focus more on the interpersonal and experiential aspects of care. Second, differences in insurance coverage and service access play a crucial role. In China’s tiered healthcare system, free medical services (often associated with specific occupational groups or public welfare programs) typically offer broader coverage, lower co-payments, and sometimes prioritized administrative handling. These systemic advantages can streamline the healthcare experience, reduce bureaucratic friction, and enhance perceived service efficiency. Out-of-pocket patients, however, navigate the standard system without such buffers, making them more vulnerable to inefficiencies and opaque pricing. Third, expectations and entitlement perceptions are shaped by payment type. Patients covered by insurance or public funds may view healthcare as an entitled service with standardized expectations, whereas out-of-pocket patients, having made direct financial commitments, may expect heightened personal attention, detailed communication, and greater flexibility. Discrepancies between these expectations and actual experiences can significantly drive satisfaction differentials.Lastly, policy and administrative integration associated with certain payment methods cannot be overlooked. Free medical services are often embedded within broader social or occupational benefit systems that may include dedicated service channels, enhanced administrative support, or more favorable treatment protocols. These “hidden” structural advantages contribute to a smoother patient journey and higher satisfaction.This finding underscores that financial mechanisms are not merely transactional but are deeply embedded in the patient’s care experience. To promote equitable satisfaction across payment groups, hospitals should enhance cost transparency, provide clear guidance on billing and insurance processes, and offer financial counseling—particularly for out-of-pocket patients. Moreover, policy measures aimed at expanding insurance coverage and reducing out-of-pocket burdens could help mitigate satisfaction disparities rooted in payment method.

Medical technology, doctor-patient communication, environmental factors, medical processes, and medical costs significantly and positively influence inpatient satisfaction. Inpatient satisfaction in the Fifth Affiliated Hospital of Wenzhou Medical University aligns with the general satisfaction levels at domestic public tertiary hospitals. Yet, there remains potential for enhancement when compared to medical institutions that exhibit superior satisfaction rates. For example, hospitals in eastern regions typically exhibit higher satisfaction levels compared to those in central and western regions [29, 30]. This study indicated that patients’ expectations and perceptions of medical services are independent of gender, age, education level, monthly income, or occupation, yet a statistically significant difference exists concerning the method of payment for hospitalization expenses. Additionally, medical costs, doctor-patient communication, environment, medical procedures, and medical technology significantly impact inpatient satisfaction. From a broad view, the differences in inpatient satisfaction levels between domestic and overseas hospitals mostly arises from variations in healthcare systems and cultural expectations [31–33]. Furthermore, variations in hospital management tiers and patient sociodemographic attributes contribute to notable disparities in satisfaction levels. From a micro perspective, the efficacy of communication between doctors and nurses in medical services, along with the precision of hospital treatment protocols, are pivotal factors; regarding the hospital environment, factors such as ward conditions and meal quality also affect patient satisfaction; the affordability of medical costs and the accessibility of medical procedures are also significant considerations [34, 35]. These factors are highly consistent with the findings of this study.

Cultural values significantly impact patient satisfaction during hospitalization [36]. The score for the doctor-patient communication dimension in this survey was 4.17 ± 0.815. Effective medical communication can cultivate trust among doctors, nurses, and patients [37], facilitating positive interactions and information exchange between healthcare professionals and patients. In clinical practice, patients often exhibit anxiety, concern, and discomfort during hospital visits, stemming from the unpredictability of disease progression and prognosis [38]. Currently, communication skills in diagnosis, treatment, and nursing are of paramount importance. Unprofessional conduct or disregard for patients’ emotions may diminish their faith in healthcare providers and can incite confrontations between physicians and patients.

An optimal hospital environment is essential for safeguarding the physical and mental health of patients, with environmental services serving as a critical indicator of hospital management quality. Environmental services encompass hospital hygiene and cleanliness, signage clarity, nutritional quality and palatability of cafeteria meals, and the adequacy of service facilities. Hospitals need to strengthen environmental management and planning to provide patients with a better medical experience.

A high score in medical procedure dimension indicates that the hospital has well-structured medical protocols that efficiently navigate patients through each stage of the process, including registration, diagnosis, examination, treatment, and medication pickup. The discharge and admission procedures are streamlined, minimizing superfluous waiting and travel time for patients and their families. The hospital provides clear medical guidance to patients, encompassing diagnostic procedures, examination components, and medication instructions, facilitating a seamless medical experience. The medical procedure dimension reflects the hospital’s present service quality and highlights opportunities for future enhancement and advancement. By persistently refining medical procedures, enhancing service efficiency, and improving patient experience, the hospital may maintain its competitive advantage in the fiercely contested healthcare industry and cultivate consumers’ confidence and loyalty.

This study revealed a medical technology satisfaction score of 4.35 ± 0.726, the highest among all dimensions, suggesting the pivotal significance of medical technology in the overall quality of hospital services. Medical technology includes a wide range of elements, comprising innovative medical devices, professional expertise, clinical experience, and the collaborative capacities of healthcare professionals. These components interconnect to create an integrated medical technology system. The expertise and proficiency of physicians, the complexity of hospital pain management protocols, and the precision and lucidity of treatment regimens significantly influence patient satisfaction.

Medical expenses received the lowest rating across all dimensions. Patients prioritize the transparency of medical charges, the fairness of drug and examination fees, and the clarity of the cost breakdown when seeking medical care. These elements directly or indirectly affect patients’ comprehensive assessment and satisfaction with hospital treatment.

This study has several implications. Hospitals should prioritize the establishment of a patient-centered service system [39], enhance training in service concepts for medical personnel, integrate fundamental medical knowledge with friendly service skills [40], and consistently elevate professional competence and service standards. Hospitals also should enhance patient feedback mechanisms by instituting suggestion boxes, launching service hotlines, and creating online evaluation platforms to collect patient opinions through multiple channels, as well as implementing a rapid response mechanism to promptly analyze and adopt feasible recommendations from patients and continually refine service processes, to deliver superior, more personalized medical care. In terms of the medical environment, it is imperative to prioritize improving the quality of patient room environments. This can be achieved by enhancing lighting and ventilation conditions, strengthening hygiene and cleaning management, and implementing infection control measures to meaningfully enhance patients’ hospital stay experience [41]. Emphasis should also be placed on the quality of dietary services, offering diverse and nutritionally balanced meal alternatives to accommodate the dietary requirements of different patients [42, 43]. Additionally, the hospital’s signage system requires enhancement to provide clear and succinct directional instructions, allowing patients and their families to quickly locate target areas. A well-thought-out spatial layout and robust infrastructure maintenance are crucial for creating a safe, comfortable, and convenient medical environment for patients. In addition to these foundational steps, hospitals can significantly improve triage efficiency by implementing an intelligent admission system [44] that integrates appointment scheduling, registration processes, and payment functions through advanced medical information technology. Investing in self-service kiosks is another key move, enabling a shift from reactive assistance to anticipatory service delivery [45] and thereby streamlining patient workflows. Furthermore, hospitals should prioritize the acquisition of advanced medical technologies to enhance diagnostic accuracy and therapeutic efficacy. This should be accompanied by the establishment of comprehensive training programs to ensure that medical staff are proficient in applying these emerging technologies [46], which will ultimately enhance patient care experiences. To ensure financial transparency, hospitals must implement real-time cost tracking systems through verified digital channels, providing patients with immediate access to detailed treatment expenditures [47]. Standardized physician-patient communication protocols should also be established, requiring clinicians to comprehensively explain both the clinical rationale for medications and their associated costs during prescription. To further bolster patients’ understanding of medical reimbursement policies [48], hospitals should establish dedicated insurance consultation counters and conduct professional education programs. Lastly, collaborating with social welfare organizations to set up medical assistance funds can offer much-needed financial support and fee reductions for economically disadvantaged patients.

There are also limitations worth noting. First, as a single-center study conducted in one tertiary public hospital in Zhejiang Province, the findings may not be fully generalizable to other regions, healthcare facility types (e.g., secondary hospitals, specialized centers), or populations with markedly different demographic or socioeconomic profiles. While the study hospital is representative of mixed urban-rural tertiary centers in eastern China, caution should be exercised when extrapolating the results to settings with distinct healthcare systems, resource levels, or patient expectations. Future multicenter studies encompassing diverse geographical and institutional contexts are needed to enhance the representativeness and national applicability of the findings. Second, the study employed a cross-sectional design without longitudinal follow-up data, making it difficult to elucidate the dynamic patterns of patient satisfaction over time. Third, because length of stay was employed as an inclusion criterion rather than a measured variable and proxy use was recorded primarily as an administrative note without being operationalized as an analytical variable, their potential roles as confounders could not be assessed. Fourth, the study methodology exclusively utilized quantitative analysis and did not incorporate qualitative research to explore patient experiences in depth, which may have overlooked other factors related to patient experience. Fifth, detailed disease type data were not collected, and ward category was used only as a sampling stratification variable rather than an analytical predictor, which prevented the assessment of condition- or ward-specific effects on satisfaction. Finally, the study did not conduct specific analyses for patients from different cultural backgrounds, which may affect the cross-cultural applicability of the conclusions.

## Conclusion

Patient satisfaction during hospitalization is one of the key indicators for assessing the quality of healthcare services, directly impacting public fulfillment and happiness. Understanding and improving patient satisfaction is of great significance for advancing the quality of healthcare institutions. This study is based on questionnaire survey data collected from inpatients at the Fifth Affiliated Hospital of Wenzhou Medical University between October and November 2024. Descriptive statistical analysis, rank sum tests, and ordered logistic regression methods were used to explore the key factors affecting inpatient satisfaction. The study found that patient satisfaction during hospitalization is influenced by six key factors: doctor-patient communication, medical technology, hospital environment, medical processes, and medical costs. It is important to note that the specific manifestations of these influencing factors may vary depending on the characteristics of the study population, regional differences, and cultural backgrounds. Healthcare facilities should concentrate on these pivotal influencing aspects to create focused quality improvement programs, to methodically elevate the quality of healthcare services and patient satisfaction.

## Supplementary Information

Below is the link to the electronic supplementary material.

Supplementary Material 1

Supplementary Material 2

Supplementary Material 3

Supplementary Material 4

## Data Availability

The de-identified datasets are available upon request.
