# Supplementary material for: Patient satisfaction and its influencing factors: results from a survey in inpatient department in a tertiary hospital setting in China
Source: BMC Health Serv Res. 2026 Feb 25;26:436. doi: 10.1186/s12913-026-14238-2 (PMC13040733; doi:10.1186/s12913-026-14238-2)
Supplement: Supplementary file 2 — Supplementary Material 2 [file 12913_2026_14238_MOESM2_ESM.pdf]

Patient Satisfaction Survey for Inpatients at The Fifth Affiliated Hospital of Wenzhou Medical University

Dear Sir/Madam,

Thank you for trusting us with your care. Our hospital is currently conducting an inpatient satisfaction survey. We sincerely hope you can take a few minutes to fill out this feedback form objectively and fairly. Your input will help us make timely improvements and enhance our services. Your support is greatly appreciated.

Informed Consent: This is an anonymous questionnaire. No sensitive personal information will be disclosed. Your answers simply represent your personal feelings—there are no right or wrong responses. Please try to be as truthful and objective as possible. The collected data will be used for academic research purposes only, and all personal information will be kept strictly confidential. By proceeding with and submitting this questionnaire, you indicate that you have understood the above information and voluntarily agree to participate in this survey.Thank you again for your assistance!

1. What is your gender?

A male   B female

2. What is your age?

A ≤20   B 20-29   C 30-39   D 40-49   E 50-59   F ≥60

3.What is your educational background?

A Middle school or below   B High school or secondary school

C Bachelor's degree or college diploma   D Master's degree or higher

4. What is your family's monthly income?

A ≤2000 yuan   B 2001-4000 yuan   C 4001-6000 yuan   D 6001-8000 yuan

E ≥8001 yuan

5. What is your occupation?

A Students   B Corporate/ institutional employees   C Workers

D Self-employed individuals   E Farmers   F Others

6. What is your payment method for this hospitalization?

A Out-of-pocket B Basic health insurance C Free medical service

7. Are you satisfied with the level of expertise and skills of the doctor?

A Very Dissatisfied B Somewhat Dissatisfied C Neutral D Somewhat Satisfied E Very Satisfied

8. Are you satisfied with the pain management measures in the hospital?

A Very Dissatisfied B Somewhat Dissatisfied C Neutral D Somewhat Satisfied E Very Satisfied

9. Are you satisfied with the advanced and complete medical equipment in the hospital?

A Very Dissatisfied B Somewhat Dissatisfied C Neutral D Somewhat Satisfied E Very Satisfied

10. Are you satisfied with the treatment plan of the hospital?

A Very Dissatisfied B Somewhat Dissatisfied C Neutral D Somewhat Satisfied E Very Satisfied

11. Are you satisfied with the clarity of the doctor's explanation of the condition and treatment plan?

A Very Dissatisfied B Somewhat Dissatisfied C Neutral D Somewhat Satisfied E Very Satisfied

12. Are you satisfied with the empathy and care shown by the medical staff in communication?

A Very Dissatisfied B Somewhat Dissatisfied C Neutral D Somewhat Satisfied E Very Satisfied

13. Are you satisfied with the ability of medical staff to use plain language in communication?

A Very Dissatisfied B Somewhat Dissatisfied C Neutral D Somewhat Satisfied E Very Satisfied

14. During your treatment, do you feel that the medical staff have fully considered your wishes and choices?

A Very Dissatisfied B Somewhat Dissatisfied C Neutral D Somewhat Satisfied

E Very Satisfied

15. Do you think the medical staff will listen to you carefully and attentively during the communication process?

A Very Dissatisfied B Somewhat Dissatisfied C Neutral D Somewhat Satisfied

E Very Satisfied

16. Are you satisfied with the service attitude of the medical staff?

A Very Dissatisfied B Somewhat Dissatisfied C Neutral D Somewhat Satisfied

E Very Satisfied

17. Are you satisfied with the clarity of the signs and directions in the hospital?

A Very Dissatisfied B Somewhat Dissatisfied C Neutral D Somewhat Satisfied

E Very Satisfied

18. Are you satisfied with the food provided by the hospital canteen?

A Very Dissatisfied B Somewhat Dissatisfied C Neutral D Somewhat Satisfied

E Very Satisfied

19. Are you satisfied with the cleanliness of the hospital?

A Very Dissatisfied B Somewhat Dissatisfied C Neutral D Somewhat Satisfied

E Very Satisfied

20. Are you satisfied with the quiet and comfortable atmosphere in the ward at night?

A Very Dissatisfied B Somewhat Dissatisfied C Neutral D Somewhat Satisfied

E Very Satisfied

21. Are you satisfied with the overall impression of the hospital service facilities (such as seats, elevators, drinking water equipment)?

A Very Dissatisfied B Somewhat Dissatisfied C Neutral D Somewhat Satisfied

E Very Satisfied

22. Are you satisfied with the efficiency of admission and discharge procedures?

A Very Dissatisfied B Somewhat Dissatisfied C Neutral D Somewhat Satisfied

E Very Satisfied

23. Are you satisfied with the process of medical services provided by the hospital?

A Very Dissatisfied B Somewhat Dissatisfied C Neutral D Somewhat Satisfied

E Very Satisfied

24. Are you satisfied with the waiting time for the medicine queue?

A Very Dissatisfied B Somewhat Dissatisfied C Neutral D Somewhat Satisfied

E Very Satisfied

25. Are you satisfied with the order of waiting in line for medical treatment?

A Very Dissatisfied B Somewhat Dissatisfied C Neutral D Somewhat Satisfied

E Very Satisfied

26. Are you satisfied with the transparency of hospital medical costs?

A Very Dissatisfied B Somewhat Dissatisfied C Neutral D Somewhat Satisfied

E Very Satisfied

27. Are you satisfied with the rationality of hospital drug costs?

A Very Dissatisfied B Somewhat Dissatisfied C Neutral D Somewhat Satisfied

E Very Satisfied

28. Are you satisfied with the reasonableness of the examination costs (e.g., CT, MRI) during your hospitalization?

A Very Dissatisfied B Somewhat Dissatisfied C Neutral D Somewhat Satisfied

E Very Satisfied

29. Are you satisfied with the clarity of the hospital's cost list?

A Very Dissatisfied B Somewhat Dissatisfied C Neutral D Somewhat Satisfied

E Very Satisfied

30. Overall, are you satisfied with the hospital?

A Very Dissatisfied B Somewhat Dissatisfied C Neutral D Somewhat Satisfied

E Very Satisfied
