# Supplementary material for: Patient satisfaction and its influencing factors: results from a survey in inpatient department in a tertiary hospital setting in China
Source: BMC Health Serv Res. 2026 Feb 25;26:436. doi: 10.1186/s12913-026-14238-2 (PMC13040733; doi:10.1186/s12913-026-14238-2)
Supplement: Supplementary file 3 — Supplementary Material 3 [file 12913_2026_14238_MOESM3_ESM.docx]

**Supplementary Table S1. Operationalization of Theoretical Frameworks into Questionnaire Items**

| ****Theoretical Framework**** | ****Core Construct / Dimension**** | ****Operationalization in the Survey**** | ****Corresponding Question(s)**** | ****Constructed Service Dimension**** |
| --- | --- | --- | --- | --- |
| ****Maslow's Hierarchy of Needs**** | \| **Physiological Needs** \| \| --- \| | Quality of basic sustenance (food) during hospitalization. | Q18 | **Environment** |
|  | **Safety Needs** | \| Perceptions of a clean, safe, and orderly environment (hygiene, noise, queuing order). \| \| --- \| | Q19, Q20, Q25 | **Environment,**  **Medical Processes** |
|  | \| **Love/Belonging & Esteem Needs** \| \| --- \| | Feeling respected, heard, and involved in care decisions (empathy, communication, consideration). | Q12, Q13, Q14, Q15, Q16 | **Doctor-Patient Communication** |
| ****Three-Dimensional Quality Structure Model (Donabedian)**** | **Structure** | Evaluation of tangible resources and facilities (equipment, signage, facilities). | Q9, Q17, Q21 | **Medical Technology,**  **Environment** |
|  | **Process** | Evaluation of interactions and procedures during care delivery (expertise, communication, efficiency). | Q7, Q8, Q10, Q11, Q12-Q16, Q22-Q25 | **Medical Technology**, **Doctor-Patient Communication**, **Medical Processes** |
|  | \| Outcome \| \| --- \| | Overall assessment of the care experience and satisfaction. | Q30 | \| **Overall Satisfaction** \| \| --- \| |
| ****Service Quality Theory (SERVQUAL)**** | **Tangibles** | Appearance of physical facilities and equipment (equipment, signage, cleanliness, facilities). | Q9, Q17, Q19, Q21 | **Medical Technology**, **Environment** |
|  | **Reliability** | Dependable and accurate service performance (expertise, pain management, treatment plans). | Q7, Q8, Q10, Q23 | **Medical Technology**, **Medical Processes** |
|  | \| **Responsiveness** \| \| --- \| | Willingness to help and provide prompt service (admission/discharge efficiency, waiting times). | Q22, Q24, Q25 | **Medical Processes** |
|  | **Assurance** | Knowledge, courtesy, and ability to inspire trust (expertise, clear  explanations, attitude). | Q7, Q11, Q16 | **Medical Technology**, **Doctor-Patient Communication** |
|  | **Empathy** | Caring, individualized attention (empathy, plain language, listening, considering wishes). | Q12, Q13, Q14, Q15 | **Doctor-Patient Communication** |
| **N/A** | \| **Cost Transparency / Financial Fairness** \| \| --- \| | Perceived fairness and clarity of medical expenses (an essential, context-specific dimension  in healthcare). | Q26,Q27, Q28, Q29 | \| **Medical**  **Expenses** \| \| --- \| |
