# Supplementary material for: Patient satisfaction and its influencing factors: results from a survey in inpatient department in a tertiary hospital setting in China
Source: BMC Health Serv Res. 2026 Feb 25;26:436. doi: 10.1186/s12913-026-14238-2 (PMC13040733; doi:10.1186/s12913-026-14238-2)
Supplement: Supplementary file 4 — Supplementary Material 4 [file 12913_2026_14238_MOESM4_ESM.docx]

**Supplementary Files**

The following supplementary files are available with this manuscript.

Supplementary File 1:The Inpatient Satisfaction Questionnaire used in this study.

**Table Legend**

| **Table 1 : Basic information of hospitalized patients and results of Mann-Whitney U/Kruskal-Wallis *H tests*** | This table presents the distribution of patients across sociodemographic groups, the proportion in each group, their corresponding mean satisfaction scores (± standard deviation), and the results of non-parametric tests assessing differences in satisfaction. The Mann-Whitney U test was used for binary variables (e.g., sex), while the Kruskal-Wallis H test was applied to variables with more than two categories (e.g., age, education level, income, occupation, and payment methods). A p-value less than 0.05 indicates statistical significance. |
| --- | --- |
| **Table 2 : Spearman Correlation Analysis of Patient Satisfaction Dimensions** | This table presents the correlation results between each dimension of medical service quality and the satisfaction of inpatients (e.g., medical technology, patient-doctor communication, environmental, medical process, and medical expenses). Spearman correlation analysis was used to evaluate the relationship between influencing factors and the satisfaction of inpatients. A p-value less than 0.05 indicates statistical significance. |
| **Table 3 : Logistic Regression Analysis of Inpatient Satisfaction Factors** | This table uses ordered logistic regression to analyze the relevant factors influencing the satisfaction of inpatients (e.g., medical technology, patient-doctor communication, environmental, medical process, medical expenses). This table includes the regression coefficient(β), odds ratio (OR), average satisfaction score (± standard deviation), multicollinearity diagnosis (VIF), and the test results for evaluating the differences in satisfaction. A p-value less than 0.05 indicates statistical significance. |

|  | **Item No** | **Recommendation** |
| --- | --- | --- |
| **Title and abstract** | 1 | (*a*) Cross-sectional Study |
|  |  | (*b*) From October to November 2024, a stratified random sampling method was adopted to conduct a questionnaire survey among 433 inpatients in the Fifth Affiliated Hospital of Wenzhou Medical University, providing a scientific basis for improving the quality of medical services and enhancing the satisfaction of inpatients. The data were analyzed using the Kruskal-Wallis test, the Mann-Whitney test and ordered logistic regression. The results show that the payment method is significantly correlated with the overall satisfaction. Ordered logistic regression analysis showed that medical technology, doctor-patient communication, environment, medical process and medical expenses were all factors affecting the satisfaction of inpatients. The overall satisfaction of inpatient services is influenced by multiple factors. The satisfaction of inpatient services can be further enhanced through measures such as optimizing doctor-patient communication, improving the hospital environment and facilities, and standardizing medical service processes. |
| Introduction | | |
| Background/rationale | 2 | The satisfaction of inpatients is the subjective evaluation of medical services by patients and an important indicator for measuring the quality of medical services. At present, there is a lack of a unified satisfaction evaluation standard system. The influencing factors of inpatient satisfaction are not clear, and the quality of medical services needs to be improved. / SERVQUAL Theory: Service quality is divided into five dimensions, including physical facilities, reliability, responsiveness, security, and emotional investment, and satisfaction is evaluated through quantitative scoring. Satisfaction not only encompasses the comprehensiveness, safety, timeliness and effectiveness of diagnosis and treatment, but also includes the quality aspect reflected throughout the entire process of medical services provided by medical staff to patients. It is a comprehensive manifestation of hospitals ensuring patients' health conditions and satisfying them. |
| Objectives | 3 | Verify the relationship between sociodemographic characteristics and satisfaction. Combined with multi-dimensional influencing factors (e.g., medical technology, patient-doctor communication, environmental, medical process, and medical expenses), propose improvement measures for the relevant factors to enhance the quality of medical services. |
| Methods | | |
| Study design | 4 | Cross-sectional study: Questionnaires were distributed to inpatients in the Fifth Affiliated Hospital of Wenzhou Medical University for a questionnaire survey to analyze the relationship between patient satisfaction and influencing factors. |
| Setting | 5 | From October to November 2024, questionnaires were distributed to inpatients in different wards of the inpatient department of the Fifth Affiliated Hospital of Wenzhou Medical University to collect data on patient satisfaction. |
| Participants | 6 | (*a*)By using the stratified sampling method, patients were randomly selected from different wards of the inpatient department of the hospital for questionnaire distribution. The criteria for patients included in the satisfaction survey are: (1) Hospitalization for more than 3 days; (2) Informed consent and voluntary participation in the investigation. |
| Variables | 7 | Independent variables: sociodemographic characteristics of inpatients(e.g., age, education level, income, occupation, sex, and payment methods), influencing factors of inpatient satisfaction (e.g., medical technology, patient-doctor communication, environmental, medical process, and medical expenses). Dependent variable: satisfaction of inpatients. |
| Data sources/ measurement | 8 | The Mann-Whitney U test was used for binary variables (e.g., sex), while the Kruskal-Wallis H test was applied to variables with more than two categories (e.g., age, education level, income, occupation, and payment methods). Ordered logistic regression was used to analyze the relevant factors influencing the satisfaction of inpatients (e.g., medical technology, patient-doctor communication, environmental, medical process, and medical expenses). |
| Bias | 9 | In terms of the content of the questionnaire, avoid leading questions. Conduct a pre-survey before the formal investigation to verify the rationality of the dimension division of the questionnaire; In terms of sample selection, it is stratified by department and ward to ensure the representativeness of the samples. |
| Study size | 10 | The sample size was calculated using statistical formulas. Considering the existence of invalid questionnaires, a total of 480 questionnaires were distributed. After data processing and eliminating invalid questionnaires, there were a total of 433. |
| Quantitative variables | 11 | The overall satisfaction was measured using the Likert five-level scale: "1" indicates "very dissatisfied" to "5" indicates "very satisfied", and the satisfaction of each dimension was evaluated using the five-level scale. |
| Statistical methods | 12 | Reliability and validity tests, confirmatory factor analysis, correlation analysis, mann-whitney u tests, kruskal-wallis h tests, ordered logistic regression. |
| Results | | |
| Participants | 13 | (a) The number of people in the pre-survey is 50. The number of people included in the study was 480. The number of people who were finally analyzed was 433. |
| Descriptive data | 14 | (a) Sociodemographic characteristics of inpatients(e.g., age, education level, income, occupation, sex, and payment methods). |
|  |  | (b) The number of participants with missing data was 47. |
| Outcome data | 15 | In terms of overall satisfaction, 297 people chose "very satisfied", 78 people chose "fairly satisfied", 34 people chose "average", 23 people chose "fairly dissatisfied", and 1 person chose "very dissatisfied". |
| Main results | 16 | (a) Medical technology(β = 0.481, OR = 1.618, P = 0.026, satisfaction score = 4.37 + 0.761); patient-doctor commumication(β = 0.876, OR = 2.401, P = 0.000, satisfaction score = 4.17 + 0.815);  environmental(β = 1.103, OR = 3.014, P = 0.000, satisfaction score = 4.11 + 0.841); medical process(β = 0.621, OR = 1.861, P = 0.001, satisfaction score = 4.35 + 0.726); medical expenses(β = 0.707, OR = 2.028, P = 0.000, satisfaction score = 4.04 + 0.911). |
|  |  | (*b*) 1 points: very dissatisfied, 2 points: relatively dissatisfied, 3 points: average, 4 points: relatively satisfied, 5 points: very satisfied. |
| Other analyses | 17 | spearman was used for correlation analysis, and it was concluded that the overall satisfaction of patients showed a significant positive correlation with each dimension of medical service quality. |
| Discussion | | |
| Key results | 18 | The gender, age, educational background, monthly income and occupation of the patients had no significant influence on the overall satisfaction. There was a significant association between the payment method of hospitalization expenses and the overall satisfaction. The overall satisfaction of inpatients is relatively good and is influenced by multiple factors. Measures can be taken to further enhance the satisfaction of inpatients from aspects such as optimizing doctor-patient communication, improving medical technology, perfecting the configuration of hospital environment and facilities, and standardizing medical service processes. |
| Limitations | 19 | Patients may be reluctant to fill out the questionnaire due to advanced age or low educational level. Temporary factors such as pain and fatigue may not reflect the true experience of medical service quality. |
| Interpretation | 20 | If the patient is unwilling to fill out the questionnaire due to old age or low educational level, the investigator will explain the content of the questions and fill them out according to the patient's oral answers. Alternatively, family members or caregivers can fill them out on behalf of the patient. Before distributing the questionnaires, explain to the patients that this survey is for the subsequent improvement of medical services in order to provide them with a better experience, and try to fill them out according to their real experiences. |
| Generalisability | 21 | The satisfaction survey focuses on the basic and general links in medical services, and these links are highly similar among different hospitals and departments. In addition, the questionnaire design refers to the satisfaction survey indicators of the performance assessment of national tertiary public hospitals, conforms to the requirements of national policies, and is applicable to most hospitals. |
| Other information | | |
| Funding | 22 | No funding was available for this study. |
